# A Practical Perspective on Confronting the Obesity Epidemic: Public-Health Strategies

**DOI:** 10.3390/nu18162602

**Published:** 2026-08-09

**Authors:** Anna Celina Owczarczyk-Durma, Leszek Czupryniak

**Affiliations:** Department of Diabetology and Internal Medicine, Medical University of Warsaw, 02-091 Warsaw, Poland; leszek.czupryniak@wum.edu.pl

**Keywords:** obesity, public health policy, sugar tax, food advertising, school food environment, front-of-pack labelling, built environment, food reformulation, bariatric surgery, GLP-1 receptor agonists

## Abstract

Obesity is currently recognized as a global epidemic and contributes to numerous health complications. It is strongly associated with poor dietary habits, including the widespread availability and consumption of high-calorie foods. We conducted a narrative review of publications from major biomedical databases addressing obesity treatment and public-health interventions. One of the strategies for managing obesity is lifestyle modification—balanced diet and regular physical activity—supported by pharmacological and psychological treatment, and, in selected patients, metabolic and bariatric surgery. Beyond patient-level care, public-health-oriented measures should be considered, including taxation of sugar-sweetened beverages, restriction on advertising of unhealthy foods, regulation of the school food environment, front-of-pack nutrition labelling, mandatory food reformulation, subsidies and incentives for healthy foods, urban-planning policies that promote physical activity, and policies that expand access to bariatric surgery. A comprehensive approach integrating clinical care with public-health policy is likely to yield more effective outcomes than either alone.

## 1. Introduction

Obesity is clinically characterised by an excessive accumulation of adipose tissue that adversely affects overall health. Obesity is considered a non-communicable modern epidemic. In 2022, over one billion people were diagnosed with obesity, representing approximately 13% of the population. It is estimated that by 2035, approximately 1.9 billion adults will be living with obesity, representing about 25% of the global population [1]. Obesity contributes to systemic inflammation and has a detrimental impact on virtually every organ. Individuals with obesity have an increased risk of multiple comorbidities, including cardiovascular disease, type 2 diabetes mellitus, and several malignancies (such as thyroid, cervical, breast and colorectal cancer), together with elevated all-cause mortality [2]. Obesity is commonly classified according to body mass index (BMI): class I corresponds to a BMI of 30.0–34.9 kg/m^2^, class II to 35.0–39.9 kg/m^2^, and class III—frequently designated “morbid obesity”—to a BMI ≥ 40.0 kg/m^2^. Individuals with a BMI between 30.0 and 34.9 kg/m^2^ show a median reduction in life expectancy of 2 to 4 years, which increases to 8 to 10 years for those with a BMI of 40.0–45.0 kg/m^2^—an effect comparable in magnitude to chronic tobacco use [2].

Recently, the conceptual framework of obesity has been refined. In January 2025, the Lancet Diabetes & Endocrinology Commission proposed to complement BMI with at least one direct measurement of adiposity (e.g., waist circumference, waist-to-hip ratio, or waist-to-height ratio) and to distinguish between preclinical obesity—excess adiposity with preserved organ function and an increased future risk of disease—and clinical obesity—a chronic, systemic illness in which excess adiposity already causes organ or tissue dysfunction [3]. As a chronic and progressive disease, obesity requires an approach that goes beyond strictly medical aspects to include psychological and social dimensions. Every patient with obesity should be systematically assessed for possible complications, including cancer screening, and offered psychological care [4].

Strategies for the management of obesity focus primarily on lifestyle modification—dietary intervention and increased physical activity—tailored to individual treatment goals. Treatment should be based on a holistic approach: behavioural modification, complemented by psychological support, pharmacotherapy, or bariatric surgery when indicated [5]. Pharmacological therapies include the bupropion–naltrexone combination, orlistat, phentermine with topiramate, and glucagon-like peptide-1 (GLP-1) receptor agonists (liraglutide and semaglutide), as well as the dual glucose-dependent insulinotropic polypeptide/GLP-1 (GIP/GLP-1) receptor agonist tirzepatide. Several novel agents are currently in late-stage clinical development, notably the triple GLP-1/GIP/glucagon receptor agonist retatrutide [6] and orforglipron—the first oral, small-molecule (non-peptide) GLP-1 receptor agonist to demonstrate meaningful weight loss in phase 3 trials. Ongoing research also includes SANA, a nitroalkene derivative of salicylate, which reduces hepatic steatosis and insulin resistance and exerts beneficial effects on body weight and glycaemia [7].

In addition to patient-centred strategies, public-health actions aimed at reducing calorie consumption in the general population have received increasing attention in recent years. Taxation of sugar-sweetened beverages, regulation of food advertising—particularly to children—and improvements in the nutritional standards of available food are the most promising strategies and potentially useful interventions. This review discusses several strategies for obesity management that involve public-health organisations. The aim is to identify broader approaches to obesity management that go beyond medical and dietary interventions.

## 2. Material and Methods

### Study Design

This article is a narrative review of public-health strategies for the prevention and management of obesity. Two electronic biomedical databases were searched: PubMed (MEDLINE) and Scopus. Database searches were complemented by hand-searching of the reference lists of the included articles and of recent expert reviews on obesity, dietary policy and metabolic disease.

The base term “obesity” was combined, in turn, with each of the following secondary terms: “diet”, “physical activity”, “family-based treatment”, “food advertising”, “weight-gain treatment”, “sugar tax”, “sugar-sweetened beverages”, “school cafeteria”, “school environment”, “front-of-pack labelling”, “built environment”, “trans fat”, “food reformulation”, “food subsidies”, and “bariatric surgery”. Boolean operators (AND, OR) were used to combine terms; the search was not restricted by study design at the search stage.

Articles were eligible for inclusion if they (i) addressed an aspect of obesity prevention or treatment, or related public-health policy in adults, children or adolescents; (ii) were published as original research, systematic reviews, meta-analyses, clinical practice guidelines, position statements from major medical or public-health organisations, or scoping or narrative reviews; (iii) were published in English; and (iv) were published between 2005 and 2026, with older landmark papers retained when they provided foundational evidence (for example, the historical pharmacology of weight-loss medications). Exclusion criteria were non-English-language publications, letters to the editor, conference abstracts without subsequent full publication, opinion pieces without supporting empirical evidence, and studies that did not specifically address obesity or one of the public-health domains under review. All authors screened titles and abstracts of retrieved records independently. Articles judged potentially relevant on this basis were then reviewed in full text; disagreements were resolved by discussion. From the eligible literature, the most up-to-date, methodologically robust, and clinically and policy-relevant publications were selected for inclusion in the narrative synthesis. Particular weight was given to systematic reviews, meta-analyses, clinical practice guidelines, large prospective cohort studies, and position statements from major international organisations (notably the WHO, the FDA, EASO, ASMBS/IFSO and the Lancet Diabetes & Endocrinology Commission on clinical obesity).

Findings were synthesised qualitatively and organised thematically into eleven public-health domains relevant to obesity prevention and management (Section 3).

**Figure 1:**
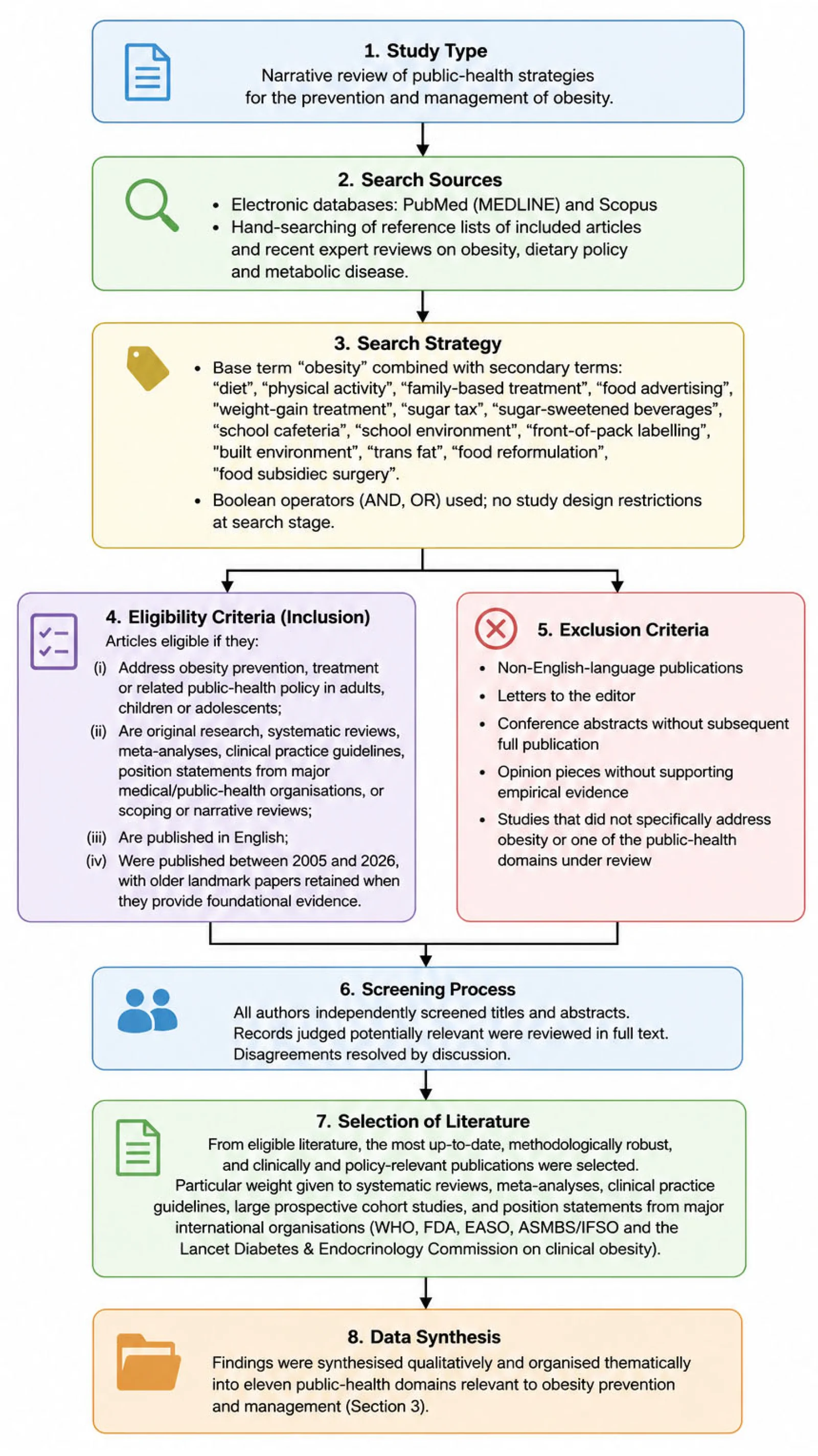
Diagram illustrating the data search process.

A summary of the search process is in the flow chart below (Figure 1)

## 3. Public-Health Strategies for Obesity Management

The cornerstone of obesity treatment is comprehensive behavioural modification—increasing physical activity and maintaining a balanced, individual diet. Special attention should be paid to patient education as to healthy lifestyle, dietary choices and physical activity, and psychological support, pharmacotherapy or metabolic surgery should be offered when appropriate. Healthcare professionals should provide this knowledge during every patient consultation; alongside such individual care, public-health initiatives play an essential role in supporting healthy lifestyles and encouraging healthy habits [8]. In this review, we discuss the following domains relevant to obesity management:

- Diet and patient-facing dietary tools;
- Pharmacological treatment;
- Taxation of sugar-sweetened beverages;
- Restriction on advertising of unhealthy foods;
- Modification of the school food environment;
- Front-of-pack nutrition labelling;
- The built environment and the promotion of physical activity;
- Food reformulation policies, including the elimination of industrially produced trans fats;
- Subsidies and financial incentives for healthy foods;
- Early-life nutrition, breastfeeding and the first 1000 days;
- Access to bariatric and metabolic surgery as a public-health issue.

### 3.1. Diet

A reduction of 5–10% of initial body weight has been shown to produce notable health benefits [9]. A proper diet is one of the most important components of obesity treatment. A dietary shift towards ultra-processed foods is observed, especially in wealthier and rapidly developing countries [10], and higher consumption of ultra-processed products is linked not only to obesity but also to its comorbidities [11]. Accurate estimation of calorie consumption is a crucial element of effective weight management; however, visually assessing the caloric content of a meal remains a challenge. Patients with obesity tend to underestimate the caloric content of meals based on visual judgement, which may itself contribute to the development of obesity [12]. This underestimation is particularly marked for meals perceived as unhealthy; Block et al. demonstrated this phenomenon for fast-food restaurants [13]. Because the process of estimating calories is often complex and time-consuming, many patients skip it entirely.

Technological advances offer promising solutions to this challenge. Recent developments in artificial intelligence have the potential to improve the accuracy of patient calorie assessment. Shonkoff et al. reported that AI-based image dietary assessment achieved accuracy for calorie and portion volume comparable to that of trained humans, with average relative errors for AI-based calorie estimation ranging from 0.1% to 38.3% [14]. Achieving a calorie deficit—and thereby promoting weight loss—requires consuming fewer calories per day than the individual daily energy requirement; understanding personal energy needs is therefore as important as the ability to assess the caloric value of meals. In a Canadian survey, 82% of respondents declared that they consider caloric value when choosing food, yet most did not know their correct daily caloric requirement; women with higher education were more likely to estimate this correctly [15]. A study by McKinnon et al. of awareness among adults in the United States found that affluent, well-educated white women were better able to estimate their caloric needs, whereas men reported a lack of awareness of their caloric requirements nearly four times more often than women [16]. Although food labelling appears to be a viable strategy for improving consumer awareness of caloric content, a study from New York did not confirm a meaningful behavioural effect: only 27.7% of respondents stated that labels influenced their purchase decisions, and no changes in calories purchased were observed compared with cities that had not introduced mandatory labelling [17].

Moreover, artificial intelligence is more often used to design individual weight-loss diets. A study by Khokhar et al. examined the impact of a digital Artificial Intelligence (AI)-based lifestyle intervention platform on weight loss. Participants lost an average of 14% of their body weight over 24 weeks. This suggests that AI-based nutritional interventions can effectively promote weight loss in patients [18]. On the other hand, a study by Kaya et al. examined the diet-planning skills of popular chatbots—Gemini, Microsoft Copilot, and ChatGPT—showing some problems of using AI. All chatbots achieved satisfactory overall diet quality. However, the diet analysis revealed an incompletely balanced distribution of macronutrients and fatty acids. Artificial intelligence-based tools appear to be gaining in importance, but they still require improvement and cannot replace professionals in the field [19].

Preventing the high prevalence of obesity in adulthood also requires reducing overweight incidence and obesity in early life [20]. Increasing awareness of the caloric contents of meals among adolescents may help. A study of 613 participants from Poland, primarily aged 14–26, found that 27% pay attention to the caloric content of meals, 42% do so occasionally, and almost one-third do not do so at all. Furthermore, 26% of respondents consider how many calories they consume daily, 43% do so occasionally, and 31% do not think about it at all; 21% do not check product ingredients when shopping [21]. A useful dietary intervention for children and adolescents is family-based treatment (FBT), in which trained therapists meet regularly with families to monitor weight loss, assess changes in habits, address current problems and set goals for the next meeting [22]. A significant barrier to implementation is the cost of specialist staff; group-based therapy could potentially provide a more scalable solution and improve nutrition among adolescents and their families.

Increased availability of food also promotes the development of obesity. The density of grocery outlets has been associated with BMI and likelihood of being overweight, with supermarkets having the greatest impact [23]. On the other hand, social-media content can influence dietary behaviour and improve eating habits [24]. Social media offer broad reach and can promote healthy eating through advice, healthy meal ideas and instruction in food-label interpretation; social-media interventions have been shown to improve population-level monitoring and behavioural control [25]. Targeted campaigns promoting a healthy lifestyle and healthy eating habits on these platforms can therefore have positive effects on weight management.

It is worth noting that dietary strategies for obesity prevention vary across income levels (low, middle, and high income) and depend on the local environment, cultural conditions, and food affordability. These strategies also vary by target group. For example, in England and France (high-income countries), policies are focused on preventing obesity from an early age, with school programs targeted at adolescents. In Mexico (a middle-income country), anti-obesity policies are more targeted at adults [26].

A summary of selected section topics is presented in Table 1.

### 3.2. Pharmacological Treatment

Pharmacotherapy is an effective intervention that should be considered alongside behavioural changes, with a focus on individual needs and preferences [27]. Over the years, the pharmaceutical market has seen the introduction and subsequent withdrawal of several weight-loss medications because of adverse effects. Historically used agents such as thyroxine and 2,4-dinitrophenol were discontinued owing to severe risks, including thyrotoxicosis and hyperthermia [28]. Similarly, amphetamine derivatives such as desoxyephedrine and diethylpropion, used to suppress appetite, were withdrawn because of a heightened risk of addiction [29]. In 1992, the efficacy of phentermine combined with fenfluramine for weight loss was reported [30]. Orlistat—which reduces intestinal fat absorption by approximately 30%—was approved and remains in clinical use [31].

Contemporary anti-obesity drugs comprise appetite-suppressing agents (such as the bupropion–naltrexone combination) and analogues of the incretin hormone GLP-1. In clinical trials, a greater reduction in body weight was observed in the bupropion–naltrexone group than in the placebo group: mean weight change was −1.3% in the placebo arm and −6.1% in the active arm [32]. Another class comprises GLP-1 mimetics; liraglutide at a maximum dose of 3 mg/day and once-weekly subcutaneous semaglutide at 2.4 mg are current standards of care. Physiologically, GLP-1 analogues mimic incretins secreted by intestinal L-cells, influencing postprandial insulin secretion [29]. A higher dose of liraglutide (3 mg vs. 1.8 mg/day) is associated with greater weight loss [29,33]. The STEP-8 randomised clinical trial showed that subcutaneous semaglutide was superior to liraglutide for weight loss in adults who were overweight or obese [34]. Dulaglutide, also a GLP-1 analogue, has a similar dose-dependent effect [35].

Tirzepatide—a dual GIP/GLP-1 receptor agonist—was approved by the US Food and Drug Administration (FDA) in May 2022 under the brand name Mounjaro for the treatment of type 2 diabetes, and on 8 November 2023 under the brand name Zepbound for chronic weight management in adults with obesity or who were overweight, plus at least one weight-related comorbidity [36]. Tirzepatide has shown effects similar to, but more pronounced than, those of GLP-1 receptor agonists alone; efficacy is dose-dependent. In December 2024, the FDA additionally approved tirzepatide for the treatment of moderate-to-severe obstructive sleep apnoea in adults with obesity, broadening its therapeutic indications.

Several other molecules are being investigated for the treatment of obesity. Retatrutide—a triple agonist of GLP-1, GIP and glucagon receptors—produced a significant reduction in body weight versus placebo in a phase 2 trial; after 48 weeks at the highest dose (12 mg/week), the mean change in body weight was −24.2% [6]. More recently, the phase 3 ATTAIN-1 trial (published in 2025) reported that orforglipron, a once-daily oral small-molecule GLP-1 receptor agonist, produced significantly greater weight loss than placebo over 72 weeks (mean change in body weight −11.2% at the 36 mg dose vs. placebo), with a safety profile consistent with the GLP-1 class [37]. As an oral, non-peptide agent that can be taken without restrictions on food or water intake, orforglipron may broaden access to pharmacotherapy if approved.

Long-term anti-obesity pharmacotherapy permits sustained weight control. After 12 months of treatment, weight reductions of 2.9–6.8% have been reported [38]. Because anti-obesity medications can cause adverse effects, treatment should always be tailored to the individual patient. Cost is a major barrier in many countries [39]; high prices mean that—despite substantial health benefits—pharmacotherapy may not be financially viable for patients when compared with intensive lifestyle intervention or Roux-en-Y gastric bypass [40]. Government support for at least partial reimbursement appears effective in mitigating the impact of obesity on public health, but is associated with considerable expenditure. The high prices of newer anti-obesity medications pose a major challenge to healthcare systems: in US Medicaid programmes, between 1999 and 2023, spending on anti-obesity medications increased dramatically, particularly with the introduction of semaglutide and tirzepatide [41]. Because of these costs, anti-obesity drug therapy is reimbursed in many countries only in selected scenarios, for example, in the presence of comorbidities (such as cardiovascular disease or dyslipidaemia). Weight regain after discontinuation of pharmacotherapy is therefore a common clinical problem driven in part by economic factors; one practical strategy is to continue with less expensive agents (e.g., bupropion–naltrexone or phentermine) after weight loss has been induced with GLP-1-based therapy [42]. Anti-obesity pharmacotherapy may also reduce long-term healthcare costs. Garvey et al. reported that anti-obesity medications were associated with improved cardiometabolic parameters (lower blood pressure and cholesterol) and, over 24 months of follow-up, fewer hospitalisations and lower medical costs [43]. Another problem is drug availability. After semaglutide was approved for the treatment of obesity, increased demand led to shortages of this drug due to insufficient supply. This also impacted the use of this drug in patients with type 2 diabetes [39].

Moreover, a survey involving healthcare workers and people with obesity identified potential barriers to the pharmacological treatment of obesity, including patients’ concerns about the effects of long-term medication use, the costs of long-term treatment, and the perception of these medications as incompatible with the treatment of chronic diseases [44].

A summary of selected section topics is presented in Table 2.

### 3.3. Taxation of Sugar-Sweetened Beverages

To reduce caloric intake and promote healthier consumer choices, several governments have introduced excise taxes on sugar-sweetened beverages. The first such tax was implemented in France in 2012; today more than 40 countries have adopted similar policies [45]. Poland introduced a tax on sugar-sweetened beverages in 2021 in order to curb the intake of high-sugar products. A 2022 analysis assessed the impact of this tax on the composition of carbonated and non-carbonated beverages. A notable reduction in sugar content was observed: from 8.6 g to 6.9 g per 100 mL in carbonated drinks, and from 5.5 g to 4.8 g per 100 mL in non-carbonated drinks, following implementation. The overall share of beverages containing more than 5 g of sugars per 100 mL fell significantly, from 70.2% in 2020 to 44.4% in 2021. After the introduction of the Polish tax, positive changes in sugar content were observed in more than half of the beverages tested [45]. Similar taxes on sugar-sweetened beverages introduced in Great Britain and Ireland in 2018 led to a fall in average sugar content, from 9.1 g/100 mL to 5.3 g/100 mL, in soft drinks [46].

Taxation of sweetened beverages may raise prices and thereby influence consumer decisions. Cabrera Escobar et al. reported that higher prices for sugar-sweetened beverages may lead to a decrease in BMI [47]. In Mexico, implementation of the tax led to an average 12% decline in sugary-drink purchases and a concomitant increase in purchases of untaxed beverages, primarily bottled water [48]. National taxation has also been associated with a slowing in the trends of overweight incidence and obesity following the intervention, with the strongest signal being among adolescents [49]. This suggests that the tax on sugar-sweetened beverages may contribute to obesity reduction over time. Despite these favourable changes in consumption patterns, there is not yet any firm evidence linking introduction of the tax with population-level reductions in body weight: the taxes appear to influence the composition of consumed meals—particularly by reducing intake of high-carbohydrate products—but their effect on average body weight in the studied populations remains inconclusive [50]. An umbrella review of the global perspective by Hajishafiee confirmed that a 10% tax on sugary drinks would reduce sugary drink consumption by 10% in high-income countries and by 9% in low- and middle-income countries. A 20% tax would reduce sugary drink consumption by 4.4 g/day in high- and middle-income countries and by 4 g/day in low- and middle-income countries [51].

### 3.4. Advertising of Unhealthy Foods

Regulating food advertising represents a potential avenue to influence and limit caloric intake. Marketing of high-fat, high-sugar and high-salt products is widely recognised for its detrimental effects on consumer health [52,53], and exposure to advertising of unhealthy foods is one of the drivers of childhood obesity [54]. Children are more susceptible to the influence of advertising than adults [55]: because of immature cognitive mechanisms, minors are unable to fully understand the intent of advertising and are therefore particularly vulnerable. By depicting the consumption of unhealthy foods alongside positive emotions and terms such as “fun”, “happiness”, “adventure” or “success”, advertisements encourage children to consume these unhealthy foods [56]. Restricting television advertising has been shown to be capable of reducing the incidence of childhood obesity [57]. Even advertising on public transport or during the school commute influences children’s consumer decisions and may contribute to obesity [58]. Cartoon characters and celebrities on the packaging of unhealthy products can also encourage children to purchase and consume them, and the placement of unhealthy foods at supermarket checkouts further influences purchasing decisions [59]. A study conducted among Indonesian teenagers (a low- and middle-income country ) confirmed that about one-third (28%) of teenagers said that ads seen on social media influenced their purchases of unhealthy foods [60].

Cumulatively, advertising of high-calorie products contributes significantly to the establishment of unhealthy eating habits from an early age.

The negative impact of unhealthy-food advertising extends to young adults. Individuals with obesity are more likely than non-obese individuals to purchase food and beverages after viewing related advertising on social-media applications (such as Snapchat, Instagram and YouTube); frequent users of these platforms also tend to consume more unhealthy foods such as fast food, crisps and sweets [61]. To curb the development of obesity, the World Health Organization (WHO) issued recommendations in 2010—most recently updated in 2023—aimed at reducing the exposure to, and power of, marketing of foods high in saturated fat, trans fat, free sugars and salt that are aimed at children. Some countries have enacted statutory regulations, but most existing rules apply only to broadcast media (radio and television) and frequently fail to address advertising on social media, posters and other outlets. In Finland, Poland and Portugal, the regulations do not cover guidelines for unhealthy-food packaging. In Canada, the regulations cover a wide range of media but leave loopholes regarding children’s magazines and entertainment events; in Chile and Australia the law applies to all commercial media [59]. The “power” of marketing is also addressed through specific techniques: in South Korea, the addition of free toys to unhealthy-food promotions has been prohibited; in Romania and Hungary, the use of cartoon characters to promote unhealthy food to children has been banned; and Poland, Sweden and the Netherlands have prohibited broadcasting of unhealthy-food advertisements during children’s programmes, as well as immediately before and after them. A related concept is food profiling—assessing fat, sugar, salt and energy content per serving—which determines whether a product is suitable for advertising. Some countries (Poland, the United Kingdom and Ireland) follow the profiling recommendations of their Ministries of Health, whereas Spain and Portugal apply WHO profiling principles. Designing universal, comprehensive legal regulation adapted to the rapidly changing advertising landscape is extremely difficult and often unachievable; nevertheless, continuing efforts to limit children’s exposure to advertising for unhealthy products remain important [59].

### 3.5. Food in the School Environment

Eating habits are formed early in life, and the surrounding environment exerts a strong influence on children’s diets. It is therefore important that the school environment fosters healthy eating habits. Some countries have introduced nutritional standards for foods and beverages available in schools, regulating calorie, sugar, saturated-fat and sodium contents. These standards have improved the quality of school products by reducing the saturated-fat and sodium contents of available snacks and by increasing fibre content [62]. A meta-analysis by Mingay et al. found that interventions in secondary schools designed to modify the nutritional profile of meals significantly improved students’ choices of fruits and vegetables. However, no single strategy was identified as clearly superior, and shorter interventions appeared to yield greater benefits—possibly because of the novelty effect on students. Common components of healthy-eating programmes include increasing the availability of healthier meals, improving the palatability of healthy food, reducing waiting times (as time for eating in schools is limited), removing highly processed foods from the school environment, and promoting healthier options through tastings, school announcements and food labelling. Greater exposure to advertising for healthy food also appears to have positive effects on children’s eating habits [63].

Kim et al. demonstrated the efficacy of promoting healthy snacks and fruit while restricting unhealthy food in school shops, coupled with promotion of healthy eating through informational brochures. In their study across nine schools, 40% of students in the intervention group purchased fruit, highlighting the positive impact of increased availability on consumption. Although overall purchasing and consumption patterns did not significantly differ between the schools compared, students in intervention schools expressed greater satisfaction with the healthy food offered [64]. Another study confirmed that modifying the product ranges offered in school cafeterias influenced children’s diets—for example, by reducing fat intake among children attending schools participating in healthy-eating programmes [65]. Introducing universal changes that cover all aspects of school nutrition (food promotion, restriction of unhealthy snacks, provision of healthy meals, and so on) is challenging, and often impossible, but progress in this sector remains worth pursuing for the sake of children’s eating habits.

The proximity of fast-food restaurants to schools also affects the development of excess body weight. Children whose schools are located within 0.5 miles (≈0.8 km) of fast-food restaurants are more likely to be overweight and to engage in unhealthy eating behaviours (such as consuming fewer fruits and vegetables) [66]. Public-health policies formally restricting fast-food outlets near schools could therefore contribute to improving children’s eating habits.

Focusing public-health policy on the nutrition of children and adolescents in schools is particularly important because eating habits established in childhood persist into adulthood [67]. A public-health policy that supports modification of children’s nutrition may therefore prevent the later development of obesity.

Moreover, the school environment should put an emphasis on physical activity, too. A systematic review by Williams et al. found that implementing a school-based policy that included education as to diet and physical activity showed promising results in reducing the prevalence of obesity and the number of those overweight. However, education that included an exclusive emphasis on physical activity in this systematic review was not associated with statistically significant decreases in BMI [65].

### 3.6. Front-of-Pack Nutrition Labelling

Information displayed on packaged foods directly influences consumer behaviour, and front-of-pack (FOP) nutrition labelling has emerged as a low-cost, population-wide intervention designed to facilitate healthier choices at the point of purchase. Interpretive FOP labels—such as the colour-coded multiple-traffic-light scheme used in the United Kingdom, the summary Nutri-Score adopted in several European countries (including France, Belgium, Germany, Spain, Switzerland, the Netherlands and Luxembourg), and the black “high-in” octagonal warning labels pioneered by Chile in 2016—have been shown in systematic reviews and meta-analyses to be more effective than purely numerical labels in improving consumer understanding of nutritional quality and in steering food choices towards healthier products [68]. Warning-label systems appear to be particularly effective. The Chilean Food Labelling and Marketing Law of 2016, which combined “high-in” octagons with parallel marketing restrictions on the same products, was followed by significant reductions in purchases of sugar-sweetened beverages and changes in consumer baskets towards less-processed alternatives [69]. FOP labelling also drives industry reformulation: manufacturers seeking to avoid a warning label tend to reduce the sugar, sodium and saturated-fat contents of their products, multiplying the health impact beyond individual consumer choice. Modelling studies suggest that, by combining behavioural change with reformulation, mandatory warning labels may meaningfully reduce the prevalence of obesity over time, although direct empirical evidence of long-term effects on population body weight remains limited. FOP labelling is a cost-effective measure that can be deployed rapidly, and its impact appears to be greatest when it is mandatory, interpretive in design, and harmonised with related policies such as marketing restrictions and excise taxes.

### 3.7. Built Environment and Promotion of Physical Activity

Obesity is an effect not only of dietary intake but also of habitual levels of physical activity. Walkable neighbourhoods, well-maintained pavements and crossings, mixed land use, access to high-quality parks and playgrounds, safe cycling infrastructure and reliable public transport all support active transport and recreational activity. A recent systematic review by Shrestha et al. confirmed that physical activity—particularly objectively measured moderate-to-vigorous activity—partially mediates the relationship between the built environment and adult obesity, with the most consistent signal being observed for composite walkability indices [70]. A separate systematic review found that interventions improving neighbourhood walkability, parks and active-transport infrastructure increased physical activity in both children and adults, although the benefits of new infrastructure may be inequitably distributed across socioeconomic groups [71]. Conversely, environmental features such as traffic-related air pollution and limited green space have been linked to higher rates of childhood obesity. Urban-planning policies that prioritise pedestrians and cyclists, distribute green spaces equitably across socioeconomic groups, and locate schools, workplaces and shops within walking distance can therefore make a meaningful contribution to population weight management—while delivering co-benefits in cardiovascular health, mental well-being, air quality and reduced traffic injuries.

### 3.8. Food Reformulation Policies and Elimination of Industrially Produced Trans Fats

In addition to taxing or labelling specific products, governments can act directly on the nutritional composition of the food supply through mandatory reformulation standards. One of the most promising strategies is the example of the elimination of industrially produced trans-fatty acids (iTFA); the World Health Organization launched the REPLACE action package in 2018, calling for global iTFA elimination by 2023, and currently recommends either a mandatory limit of 2 g of trans fat per 100 g of total fat in all foods or a ban on partially hydrogenated oils as a food ingredient [72]. Early adopters such as Denmark (which restricted iTFA in 2003) and New York City (which banned iTFA from restaurants in 2007) saw substantial reductions in cardiovascular mortality and hospitalisations following implementation, demonstrating that population-wide reformulation is both feasible and cost-effective [73]. Although the most direct health gains from iTFA elimination concern cardiovascular disease, reformulation policies illustrate how regulating the food supply can change population nutrient intake without requiring changes in individual behaviour, and the same logic now underpins efforts to reduce salt, free sugars and saturated fat in processed foods.

### 3.9. Subsidies and Financial Incentives for Healthy Foods

Whereas taxes on sugar-sweetened beverages and other ultra-processed products use price as a disincentive, the mirror-image strategy is to use price as an incentive—that is, to subsidise the consumption of fruits, vegetables and other healthy foods. A 2025 systematic review of 18 studies on healthy-food subsidies reported that subsidised fruit and vegetable purchases consistently rose during intervention periods, although the effects on body weight and BMI remained inconclusive and tended to reverse once incentives were withdrawn [74]. A meta-analysis published in The Lancet Planetary Health showed that financial incentives—particularly when targeted at low-income groups—modestly increased the purchase and consumption of healthy foods [75]. Real-world examples include enhanced Supplemental Nutrition Assistance Program (SNAP) incentives in the United States, Healthy Start vouchers in the United Kingdom, and analogous fruit-and-vegetable voucher schemes in several European countries. Subsidies are politically more palatable than taxes and address the well-documented socioeconomic gradient in obesity prevalence by reducing the cost of healthy food for households facing the greatest economic constraints. Combining “carrots” with “sticks”—pairing healthy-food subsidies with taxes on energy-dense, nutrient-poor products—appears to offer a more comprehensive policy package than either approach alone.

### 3.10. Early-Life Nutrition, Breastfeeding and the First 1000 Days

Eating habits are not the only obesity-related exposure that begins in childhood; the biological and behavioural foundations of body weight regulation are laid much earlier, during the “first 1000 days” from conception to the end of the second year of life. This window encompasses maternal pre-pregnancy weight, gestational weight gain, gestational diabetes, smoking during pregnancy, infant feeding mode, the timing and content of complementary feeding, and early growth trajectories—all of which independently and synergistically influence the risk of later overweight incidence and obesity. The recent narrative review by the EU-funded EndObesity Consortium synthesised evidence from large multi-cohort studies and identified maternal pre-pregnancy obesity, excess gestational weight gain, accelerated infant weight gain and inappropriately early introduction of solid food as key modifiable risk factors for childhood obesity, calling for coordinated preconception, prenatal and postnatal interventions delivered through primary-care and public-health systems [76].

Promotion and protection of breastfeeding is the most evidence-based component of this strategy. The most recent systematic review and meta-analysis commissioned by the WHO included 159 studies and 169 effect estimates and reported a pooled odds ratio of 0.73 (95% confidence interval 0.71–0.76) for the protective effect of breastfeeding on later overweight incidence or obesity; the association persisted, with attenuation, even when analyses were restricted to studies less susceptible to publication bias and confounding [77]. WHO currently recommends exclusive breastfeeding for the first six months and continued breastfeeding, with appropriate complementary foods, up to two years of age or beyond. At the population level, breastfeeding rates are powerfully influenced by policy, including paid maternity leave, baby-friendly hospital accreditation, workplace lactation support, and—critically—implementation of the WHO International Code of Marketing of Breast-milk Substitutes, which restricts the aggressive marketing of infant formula and follow-on milks [78]. Public-health systems that combine these structural measures with individual-level support tend to achieve substantially higher rates of exclusive breastfeeding and, by extension, lower long-term obesity risk in their populations. Early-life interventions are particularly attractive from a public-health perspective because they act upstream of the obesogenic environment and address the strong intergenerational transmission of obesity risk. Public-health strategies should also include support for low-income households, as well as nutritional support and counselling for parents [79].

### 3.11. Access to Bariatric and Metabolic Surgery as a Public-Health Issue

Bariatric and metabolic surgery remains one of the most effective and durable treatments for severe obesity, achieving in most patients a sustained weight loss of 20–30% of initial body weight together with marked improvements in, or remission of, type 2 diabetes, hypertension, dyslipidaemia, obstructive sleep apnoea and metabolic dysfunction-associated fatty liver disease. In 2022, the American Society for Metabolic and Bariatric Surgery (ASMBS) and the International Federation for the Surgery of Obesity and Metabolic Disorders (IFSO) jointly updated the indications for metabolic and bariatric surgery—replacing the 1991 US National Institutes of Health criteria—recommending surgery for all individuals with a body mass index (BMI) ≥ 35 kg/m^2^ regardless of comorbidities, considering it for those with BMI 30–34.9 kg/m^2^ and metabolic disease, lowering the BMI threshold for Asian populations (≥27.5 kg/m^2^), and explicitly including appropriately selected children and adolescents [80]. Long-term follow-up from the Swedish Obese Subjects study has shown that bariatric surgery, compared with conventional treatment, is associated with reductions in overall mortality, cardiovascular events, and incident type 2 diabetes and cancer [81].

Despite this evidence, the proportion of eligible patients who undergo surgery is small in most health systems, and access is highly inequitable across—and within—countries. Barriers include persistent obesity stigma in healthcare and society, restrictive reimbursement criteria, limited specialist capacity, inadequate funding of the multidisciplinary preoperative and postoperative care that good outcomes require, and the perception that conservative treatment alone is sufficient. From a public-health perspective, expanding equitable access to bariatric and metabolic surgery is therefore not merely a clinical question but a policy one: aligning national reimbursement frameworks with current evidence-based indications, funding multidisciplinary obesity centres, training the necessary workforce, integrating surgical pathways with pharmacological and behavioural treatment, and tackling weight stigma in health systems. As pharmacotherapy continues to evolve and the boundary between medical and surgical treatment shifts, integrated obesity care—in which surgery, pharmacotherapy and lifestyle intervention are coordinated rather than offered in sequence after failure of the previous step—is increasingly being proposed as the appropriate public-health model.

## 4. Conclusions

The treatment of obesity requires individualised behavioural intervention, including a tailored diet, supported when necessary by pharmacotherapy, psychological care and, in selected patients, metabolic and bariatric surgery. Because public-health policy can significantly influence the prevalence of obesity, it can be used to shape population behaviour and complement clinical treatment. A summary of the effectiveness and feasibility of individual public-health strategies, based on the authors’ expert opinion, is presented in Table 3. It is the authors’ expert interpretation of the available evidence rather than a formal evidence-based ranking (e.g., based on GRADE, Delphi consensus, or other structured methodologies). As summarised in Table 1, the range of effective tools is now considerable and spans food reformulation, front-of-pack labelling, modification of the school food environment, taxation of sugar-sweetened beverages, early-life and first-1000-days interventions, restriction of food advertising, pharmacotherapy, bariatric surgery, subsidies for healthy foods, urban-planning policies that support active transport, and individual-level dietary tools. Such measures should always be adapted to the local cultural, economic and regulatory contexts for maximum effectiveness, and are likely to work best when deployed as coordinated packages rather than in isolation. Social media appears to be a promising channel for promoting education as to healthy lifestyles, particularly in younger generations. However, it should be noted that most of the strategies discussed lack conclusive evidence for long-term weight loss. Another limitation of the present study is the relatively limited attention paid to the quality and methodology of the studies discussed in this narrative review. Another limitation of this review is the diversity of healthcare systems, socioeconomic conditions, and cultural differences in evidence in this manuscript. Direct comparisons between interventions should therefore be interpreted with caution. The proposed public-health strategies are not universal for every setting and require individualized interventions.

Future research strategies should examine the long-term effects of implemented public-health interventions. Furthermore, it would be beneficial for studies to include multifactorial effects, which would reflect the effects of interventions more realistically, such as the impacts of diet, physical activity, access to healthcare, and other factors. Another interesting future direction seems to be the development of artificial intelligence, which holds great promise for better personalizing nutritional and educational plans for weight loss.

## Figures and Tables

**Table 1:** Summary of selected topics related to diet and their impacts on public health.

Topic	Public-Health Implications	Reference(s)
Calorie estimation	Improving calorie estimation skills may facilitate more effective weight management.	[12,13]
Artificial intelligence (AI)	AI may support self-monitoring of dietary intake and improve adherence to weight-loss interventions.	[14]
Awareness of energy requirements	Nutrition education should include personalized estimation of daily energy requirements.	[15]
	Improving nutritional literacy during adolescence may contribute to obesity prevention.	[20,21]
Food labelling	Food labelling alone is unlikely to substantially change eating behaviour without complementary educational strategies.	[17]
Family-based treatment (FBT)	Group-based interventions may represent a more scalable alternative to individual FBT.	[22]
Food environment	Environmental and policy interventions are needed to promote healthier food environments.	[10,11,23]
Social-media interventions	Targeted digital health campaigns may complement conventional obesity prevention and management programs.	[24,25]

**Table 2:** Summary of selected topics related to pharmacological treatment of obesity and their impacts on public health.

Topic	Public-Health Implications	Reference(s)
Integration of pharmacotherapy into obesity management	Clinical guidelines should promote multidisciplinary, personalized obesity management rather than lifestyle intervention alone.	[27]
Innovation in obesity treatment	Continued pharmaceutical innovation has the potential to improve access, adherence, and long-term treatment outcomes.	[6,37]
Long-term disease management	Obesity should be managed as a chronic disease requiring long-term treatment strategies and follow-up.	[38,42]
Health inequalities	Policies that improve affordability and equitable reimbursement may reduce disparities in obesity care.	[40,41,42]
Healthcare expenditure	Healthcare systems must balance the budgetary impacts of new therapies with their long-term health benefits.	[41]
Economic benefits of treatment	Effective obesity treatment may generate long-term savings by reducing obesity-related morbidity and healthcare utilization.	[43]

**Table 3:** A ranking of eleven public-health strategies, based on the authors’ assessment (and representing the authors’ expert interpretation). The strategies discussed in in this review are ordered according to their combined workability and population-level effectiveness. The ranking integrates strength of the evidence base, political and economic feasibility, speed of impact, equity considerations and the scale at which each intervention can plausibly be deployed. It reflects the authors’ interpretation of the clinical and public-health perspectives relevant to the use of these strategies; alternative orderings are defensible depending on local epidemiology, health-system capacity, and policy priorities. The ranking is intended to serve as a suggested priority, not as a definitive order.

	Strategy	Workability	Effectiveness	Key Consideration
1	Food reformulation; elimination of industrially produced trans fats (WHO REPLACE)	High	High	A WHO “best-buy”; invisible to consumers; demonstrated at country scale; low political friction.
2	Front-of-pack warning labels (e.g., Chile “high-in” octagons; Nutri-Score)	High	Moderate–High	Drives industry reformulation; cheap to deploy; more politically tractable than excise taxes.
3	School food environment (nutrition standards; restrictions on UPF; healthy-meal promotion)	High	Moderate–High	Acts on a captive paediatric audience; shapes habits before they are fixed; effects strongest in younger children.
4	Taxation of sugar-sweetened beverages	Moderate–High	Moderate	Adopted by >40 countries; reduces SSB intake and drives reformulation; effect on body weight modest at population level.
5	Early-life and first-1000-days interventions; protection and promotion of breastfeeding	High	Moderate (long latency)	Strong upstream evidence; effects accrue over decades; depends on maternity leave, hospital practices and marketing restrictions.
6	Pharmacotherapy (GLP-1 receptor agonists, tirzepatide, emerging oral GLP-1s)	Low (cost-limited)	Very high at individual level	A clinical lever more than a public-health one; population reach constrained by price and reimbursement policy.
7	Equitable access to bariatric and metabolic surgery	Low–Moderate	Very high (most durable Tx)	Treats existing disease rather than preventing incident obesity; access limited by cost, stigma and specialist capacity.
8	Restriction on advertising of unhealthy foods (especially to children)	Moderate	Moderate–High in children	Effective in regulated broadcast media; large gaps in digital/social-media advertising; sustained industry opposition.
9	Subsidies and financial incentives for healthy foods	Moderate	Low–Moderate	Politically palatable; modest effect on diet quality; reverses on withdrawal; useful complement to SSB taxes.
10	Built environment; urban planning for active transport	Low (slow, capital-intensive)	Moderate (long term)	Decade-scale infrastructure lever; substantial co-benefits in cardiovascular health, mental well-being and air quality.
11	Individual-level diet tools (apps, AI-based calorie estimation, family-based therapy)	High (per person)	Low–Moderate at population level	Effective for engaged individuals; very hard to scale; high recidivism without structural support.

## Data Availability

No new data were created or analysed in this study. Data sharing is not applicable to this article.
